# Host dependent maintenance of a *bla*_NDM-1_-encoding plasmid in clinical *Escherichia coli* isolates

**DOI:** 10.1038/s41598-020-66239-8

**Published:** 2020-06-09

**Authors:** João Alves Gama, Julia Kloos, Pål J. Johnsen, Ørjan Samuelsen

**Affiliations:** 1 Department of Pharmacy, Faculty of Health Sciences, UiT The Arctic University of Norway, Tromsø, Norway; 2 Norwegian National Advisory Unit on Detection of Antimicrobial Resistance, Department of Microbiology and Infection Control, University Hospital of North Norway, Tromsø, Norway

**Keywords:** Evolution, Microbiology

## Abstract

Dissemination of bacterial clones carrying plasmid-mediated resistance genes is a major factor contributing to the increasing prevalence of antibiotic resistance. Understanding the evolution of successful clones and the association to mobile resistance elements are therefore crucial. In this study, we determined the sequence of a 145 kb IncC multi-drug resistance plasmid (pK71-77-1-NDM), harbouring resistance genes to last-resort antibiotics including carbapenems. We show that the plasmid is able to transfer into a range of genetically diverse clinical *Escherichia coli* strains and that the fitness cost imposed on the host is often low. Moreover, the plasmid is stably maintained under non-selective conditions across different genetic backgrounds. However, we also observed a lower conjugation frequency and higher fitness cost in the *E. coli* sequence type (ST) 73 background, which could partially explain why this clone is associated with a lower level of antibiotic resistance than other *E. coli* clones. This is supported by a bioinformatical analysis showing that the ST73 background harbours plasmids less frequently than the other studied *E. coli* STs. Studying the evolution of antibiotic resistance in a clinical context and in diverse genetic backgrounds improves our understanding of the variability in plasmid-host associations.

## Introduction

The development of antibiotic resistance in bacterial populations is an inevitable evolutionary consequence of the selective pressure exerted by the use and misuse of antibiotics. Two main routes allow bacterial evolution towards antibiotic resistance: mutations in the chromosome or acquisition of mobile genetic elements (MGEs) harbouring resistance-determinants.

Plasmids are extrachromosomal, independently replicating, most often circular and transferable DNA molecules that constitute the most prominent group of MGEs facilitating horizontal spread of antibiotic resistance^1^. Transferable plasmids harbouring resistance-determinants are widespread among clinically relevant Gram-negative pathogens like *Escherichia coli*^2^. This includes plasmids encoding extended-spectrum β-lactamases (ESBLs) or carbapenemases conferring resistance to β-lactams such as cephalosporins and carbapenems^3^. In *E. coli*, a wide diversity of plasmid associated ESBL and carbapenemase genes have been identified including *bla*_CTX-M_, *bla*_KPC_, *bla*_NDM_, *bla*_VIM_, *bla*_IMP_, and *bla*_OXA-48-like_^4^. Interestingly, the molecular epidemiology of *E. coli* supports that certain resistance genes are clearly linked to specific dominant *E. coli* clones and plasmid backbones like the association of *bla*_CTX-M-15_ with IncF family plasmids and sequence type (ST) 131^2^. It has been suggested that *E. coli* ST131 may dominate as a successful multi-drug resistant clone due to the ability to offset the fitness cost of plasmid acquisition and maintenance via compensatory mutations in gene regulatory regions^5,6^. In contrast, other resistance genes like *bla*_NDM_ show a more broad diversity, both with respect to host genetic backgrounds and plasmid backbones^2,7^. In addition to successful multi-drug resistant *E. coli* clones, other clones like ST73 have been shown to be equally successful, but generally susceptible to antibiotics^8,9^.

The success of a plasmid, and consequently a plasmid associated resistance gene, is constrained by several factors like conjugation rate, incompatibility with other plasmids in the same cell, stability and fitness cost^10–14^. The majority of studies investigating these mechanisms and plasmid-host interactions are performed using laboratory-adapted strains, environmental bacteria and/or plasmids with limited clinical relevance. A scarcity of studies has focused on clinically relevant pathogenic bacteria using plasmids with resistance-determinants observed in the clinical setting^15,16^. With respect to carbapenemase-encoding plasmids, it has been shown that a clinical *bla*_NDM-1_-encoding plasmid was stably maintained in laboratory strains of *E. coli* and *Klebsiella pneumoniae*, but imposed a significant fitness cost^17^. In contrast, we have previously shown that acquisition of clinical plasmids carrying *bla*_KPC-2_ or *bla*_VIM-1_ genes by plasmid-naïve clinical *E. coli* strains of different genetic backgrounds resulted in low to moderate reductions in fitness cost (1.1-3.6%) and that the fitness cost and plasmid stability were both plasmid and host dependent^18^. This relatively low impact on host fitness exerted by clinical carbapenemase-encoding plasmids has also been shown for a *bla*_KPC-2_-positive plasmid in *K. pneumoniae*^19^.

To further investigate the impact of plasmid acquisition, we studied the conjugation frequency, fitness cost and stability of a ~145 kb IncC *bla*_NDM-1_-encoding clinical plasmid in a genetically diverse collection of clinical *E. coli* strains.

## Results and discussion

### Characterization of the bla_NDM-1_ plasmid and donor strain

PacBio sequencing of the *bla*_NDM-1_-carrying *E. coli* donor strain K71-77 resulted in a circularized chromosome (4,934,660 bp) and two circularized plasmids, pK71-77-1-NDM (145,272 bp) and pK71-77-2 (117,597 bp). The genomic data confirmed that K71-77 belongs to phylogroup A and ST410^20^, which is considered an emerging international high-risk clone^21^. The isolate carried *fimH24* encoding type I fimbriae and chromosomal mutations in *gyrA* (S83L and D87N), *parC* (S80I) and *parE* (S458A) conferring fluoroquinolone resistance common to ST410^21^. No acquired resistance genes were identified on the chromosome.

**Figure 1:**
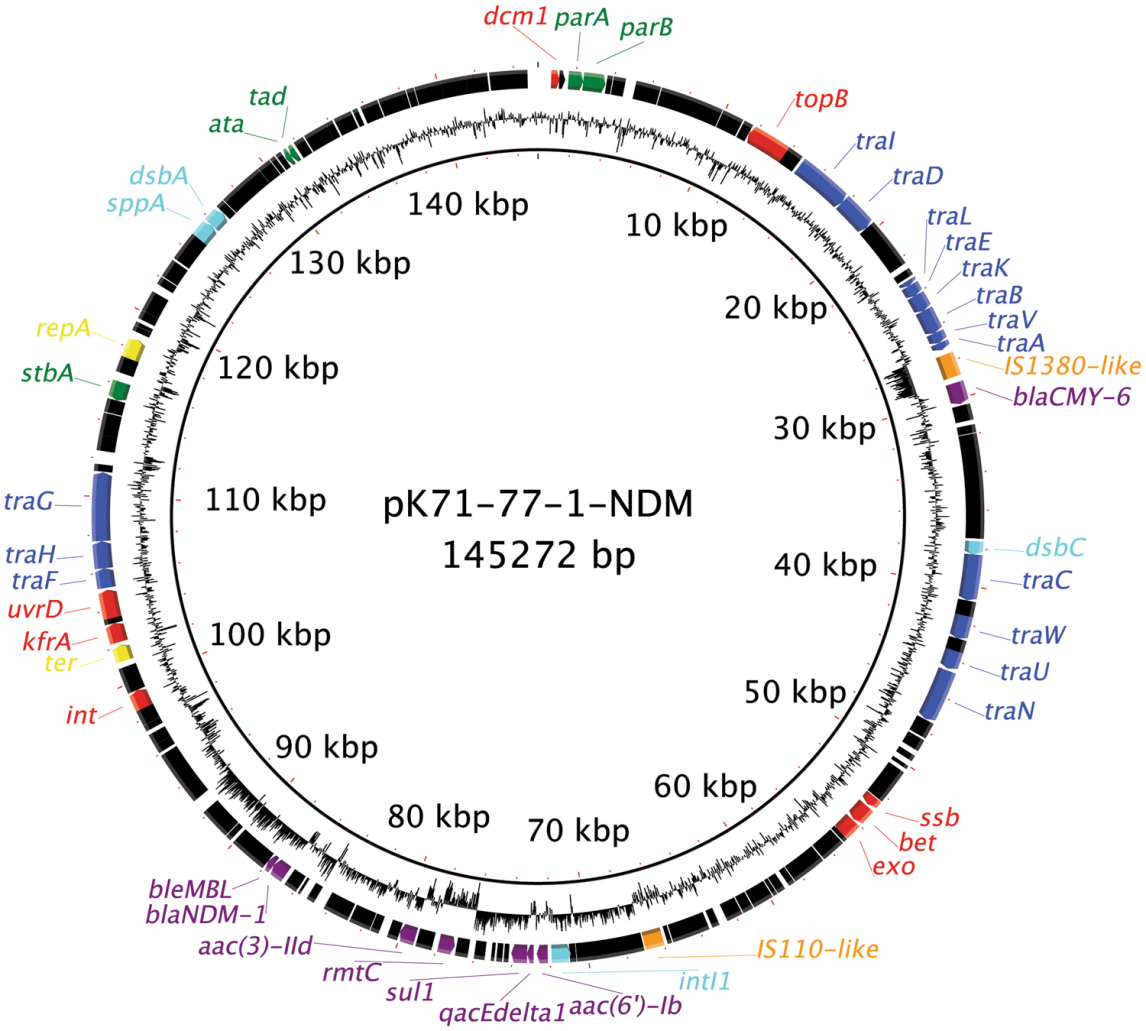
Genetic map of pK71-77-1-NDM. Specific CDSs are colour coded as follows: DNA metabolism, red; plasmid maintenance, green; conjugative transfer, blue; mobile element, orange; other, turquoise; antimicrobial resistance, purple; replication, yellow. The map was constructed using the BLAST Ring Image Generator^45^.

The *bla*_NDM-1_-carrying plasmid (pK71-77-1-NDM) is an IncC type 1 plasmid (also termed IncA/C_2_) based on the R1 and R2 regions and i1 and i2 segments^22^ as well as cgST1.2 according to the cgPMLST IncA/C scheme^23,24^. In addition to *bla*_NDM-1_, pK71-77-1-NDM carried the plasmid-mediated AmpC gene *bla*_CMY-6_, the 16S rRNA methylase gene *rmtC*, two variants of aminoglycoside acetyltransferase genes *aac(6’)-Ib* and *aac(3)-II*, the sulfonamide resistance gene *sul1* and the bleomycin resistance gene *ble*_MBL_ (Fig. 1). pK71-77-1-NDM displayed ~99% identity over its entire sequence with other IncC *bla*_NDM_-encoding plasmids like pNDM-EcoGN568 (GenBank acc. no. KJ802404), pNDM102337 (GenBank acc. no. JF714412), pNDM10505 (GenBank acc. no. JF503991) and pNDM-PstGN576 (GenBank acc. no. KJ802405). The second plasmid, pK71-77-2 is an IncF (IncFIA, IncFIB, IncFII) plasmid of replicon sequence type F36:A4:B1, encoding resistance to aminoglycosides (*aac*(*6*′)*-Ib-cr*), β-lactams (*bla*_OXA-1_) and chloramphenicol (*catB4*).

### Plasmid conjugative transfer

**Figure 2:**
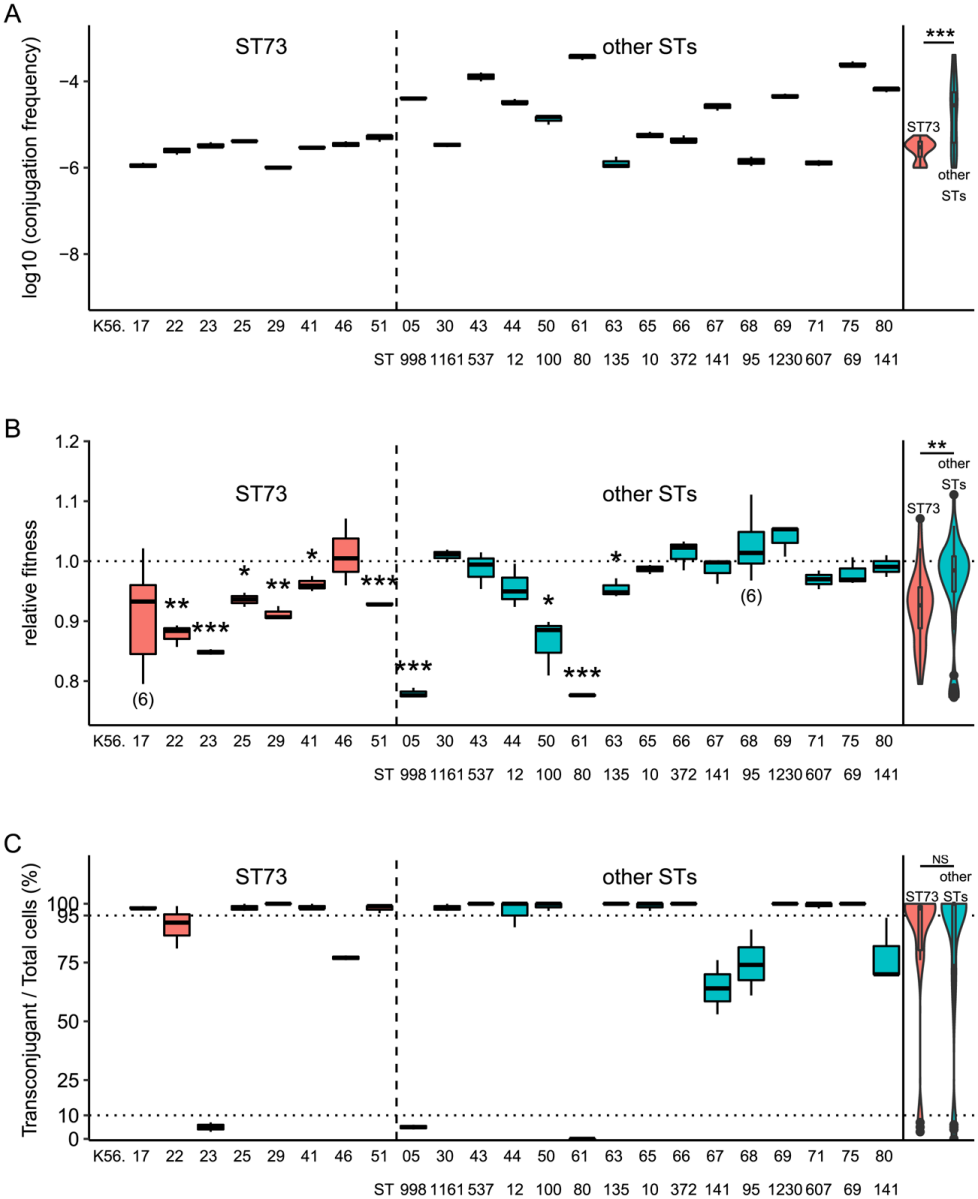
(**A**) Conjugation frequency of pK71-77-1-NDM from *E. coli* K71-77 into rif^R^ K56 ECO-SENS strains. Vertical axis indicates the log_10_ of the conjugation frequency. (**B**) Relative fitness of rif^R^ K56 ECO-SENS strains carrying pK71-77-1-NDM. Vertical axis indicates the fitness of plasmid-carrying strains relatively to their isogenic plasmid-free counterpart. (**C**) Stability of plasmid pK71-77-1-NDM in rif^R^ K56 ECO-SENS strains. Vertical axis indicates the percentage of plasmid-carrying cells in the population. For all plots the horizontal axes indicate the rif^R^ K56 strains’ suffix, and ST for non-ST73 strains; left panel: strains belonging to ST73, middle panel: strains belonging to other STs, right panel: overall comparison between ST73 and other STs. ^NS^, ** and *** denote respectively: non-significant (p value > 0.05), p value < 0.01 and p value <0.001 (Wilcoxon test). The graphic illustrations were performed in R^44^.

We mated the *E. coli* K71-77 isolate carrying pK71-77-1-NDM, in liquid for 3 hours (1:1 donor:recipient ratio), with 23 different uropathogenic recipient strains belonging to 15 different STs (Table 1). The recipient strains were selected from the ECO-SENS collection found to be devoid of phenotypic resistance to 24 antibiotics and plasmid-naïve based on plasmid-replicon typing and S1 nuclease pulsed-field gel electrophoresis^25^. This enabled us to introduce rifampicin resistance as a selection marker in the conjugation experiment and to avoid interactions with other plasmids. Plasmid pK71-77-1-NDM was successfully transferred by conjugation to all recipient strains, but with varying frequencies, ranging from 1 × 10^−6^ to 4.1 × 10^−4^ transconjugants per donor (Fig. 2A). In general, strains belonging to ST73 acquired the plasmid less efficiently than other STs (Wilcoxon test, p < 0.001; Fig. 2A). This finding corroborates the potential of IncC plasmids to spread into different genetic backgrounds and play a significant role in the dissemination of antibiotic resistance^22^. However, the results indicate that there are differences in the conjugation frequencies dependent on the genetic background as also shown for other resistance plasmids^18,26^. Thingholm *et al*. also showed that the transfer frequency of a plasmid carrying *bla*_CTX-M-15_ to ST73 was lower than for other genetic backgrounds^26^.

**Table 1:** *E. coli* strains used in the study^20,25,36^. K71-77 harbours plasmids pK71-77-1-NDM and pK71-77-2. All K56- strains are spontaneous rif^R^ mutants of the isolate in^25^.

Strain	Sequence type	Phylogroup
K71-77	ST410	A
K56-5	ST998	B2
K56-17	ST73	B2
K56-22	ST73	B2
K56-23	ST73	B2
K56-25	ST73	B2
K56-29	ST73	B2
K56-30	ST1161	B2
K56-41	ST73	B2
K56-43	ST537	B2
K56-44	ST12	B2
K56-46	ST73	B2
K56-50	ST100	A
K56-51	ST73	B2
K56-61	ST80	B2
K56-63	ST135	B2
K56-65	ST10	A
K56-66	ST372	B2
K56-67	ST141	B2
K56-68	ST95	B2
K56-69	ST1230	A
K56-71	ST607	A
K56-75	ST69	D
K56-80	ST141	B2

### Fitness effect and stability of pK71-77-1-NDM

To investigate if genetic background impacts the plasmid’s stability and fitness effect, we selected one transconjugant per strain and verified the presence of pK71-77-1-NDM by PCR (IncA/C2 replicon and *bla*_NDM-1_) and antibiotic resistance profile. Absence of the additional plasmid pK71-77-2 was confirmed by PCR for the IncFIA replicon. Host background was confirmed by RAPD-PCR-profiling and Sanger sequencing of the *fumC* allele.

We calculated exponential growth rates of the plasmid-free and plasmid-carrying strains by measuring the optical densities of monocultures as a function of time. The ratio between the growth rates of isogenic strains (differing only in the presence of pK71-77-1-NDM) served as a proxy to determine the fitness effect of plasmid carriage for each strain background. Plasmid pK71-77-1-NDM imposed detectable fitness costs varying between 2.5% to 22.7% in 10 of the 23 strains (T test, p < 0.05; Fig. 2B), mainly imposed on strains belonging to ST73 (Wilcoxon test, p = 0.002; Fig. 2B). Our data here indicate that the effect on fitness is strongly dependent on the recipient background, providing further support to previous reports^18,27–29^. The fitness cost of plasmids has been attributed to multiple factors^14^ and the mechanistic basis for this difference in plasmid cost is unclear. However, it is well known that acquisition of plasmids impacts the expression of chromosomal genes and consequently physiological processes that could cause a fitness effect^19,30^. One could therefore speculate that fitness differences upon plasmid acquisition could be due to variability in the interactions of plasmid-encoded proteins on the cellular networks in the recipients, which are likely to be distinct based on the diversity in genetic backgrounds. A detailed analysis would be required to elucidate the factors implicated in the variability of fitness effects between the different genetic backgrounds. The lack of initial fitness cost of pK71-77-1-NDM in more than half of the strains is worrying as this indicates potential for successful plasmid maintenance in different genetic backgrounds and in non-selective environments without the need for compensation. Others and we have shown that clinical plasmids frequently impose a limited fitness cost, including plasmids harbouring *bla*_NDM_^18,31–33^.

We further propagated the plasmid-carrying strains for ≈300 generations in the absence of selection to assess plasmid stability. Most strains maintained pK71-77-1-NDM stably in the population (Fig. 2C). For 14 strains, the plasmid was retained in more than 95% of the cells, while for three strains less than 10% of the cells carried pK71-77-1-NDM after 300 generations. We observed no difference in plasmid stability between strains belonging to ST73 and the other genetic backgrounds (Wilcoxon test, p > 0.05; Fig. 2C). Overall, this indicates that pK71-77-1-NDM can be stably maintained in variable genetic backgrounds under non-selective conditions further corroborating the epidemic potential of the plasmid. Like other IncC plasmids, pK71-77-1-NDM harbours a putative toxin-antitoxin system that could contribute to the maintenance of the plasmid^23,34^.

### Frequency of plasmid-carriage

ST73 is considered a major pandemic *E. coli* clone frequently shown to be the dominating clone causing urinary tract or bloodstream infections^9,35^. In a collection of pan-susceptible *E. coli*, ST73 was found to be the clone most frequently devoid of plasmids^25^. Since we observed lower conjugative transfer rates and higher plasmid fitness costs in ST73 compared to the other genetic backgrounds (Fig. 2A and B) we performed a bioinformatical approach to compare ST73 with the other STs we had tested experimentally in terms of plasmid content. We downloaded all the 14830 *E. coli* assembled genomes available in December 2018 from the NCBI GenBank (ftp://ftp.ncbi.nlm.nih.gov/genomes/genbank/bacteria/Escherichia_coli/latest_assembly_versions/). 2182 of these genomes were unambiguously classified in STs employed in our experiments, 305 of which belonged to ST73 (Table S2). Then, we used PlasmidFinder to identify the plasmid content of these genomes. We found that most genomes, 1836 out of 2182, harboured plasmids. 69% of ST73 genomes harboured plasmids, but this proportion was significantly higher, 87%, for genomes belonging to other STs (χ^2^ test, d.f. = 1, p = 6.3 × 10^−15^; Table 2). Our bioinformatic analyses of plasmid content in ST73 versus other STs are consistent with the experimental conjugation and relative fitness data suggesting that ST73, for some plasmids, appears to be a sub-optimal host.

**Table 2:** Comparison of plasmid presence/absence between ST73 and the other STs studied. ^*^Pearson’s χ^2^ test with Yates’ continuity correction.

No. (%) of genomes		ST73	Other STs	p-value^*^
Plasmids	Presence	210 (69)	1626 (87)	6.3 × 10^−15^
	Absence	95 (31)	251 (13)	

## Conclusion

We show here that the conjugation frequency and fitness impact of a multi-drug resistance plasmid carrying the carbapenemase gene *bla*_NDM-1_ is dependent on the recipient host background. Moreover the lack of, or relatively low fitness cost imposed by pK71-77-1-NDM in diverse genetic backgrounds combined with stable maintenance in a non-selective environment shows the epidemic potential of the plasmid. The plasmid’s lower conjugation frequency and higher fitness cost in ST73 could partially explain why ST73 is less associated with antibiotic resistance than other successful epidemic clones. Our study also shows the importance of investigating the evolution of antibiotic resistance in clinical strains with diverse genetic backgrounds. One limitation of the study is the representation of genetic backgrounds relative to the overall *E. coli* population. Further work is required to investigate if there are other *E. coli* lineages that show similar properties as ST73. Investigations into the mechanistic aspects of these observations will be important for possible interventions to limit the spread of antimicrobial resistance^15,16^.

## Methods

### Strain collection and whole genome sequencing

Bacterial strains used in the study and relevant characteristics are summarized in Table 1. *E. coli* K71-77 previously shown to carry *bla*_NDM-1_^20,36^ was sequenced using PacBio sequencing (Pacific Biosciences, Menlo Park, CA). Genomic DNA was isolated from an overnight culture using the GenElute bacterial genomic DNA kit (Sigma-Aldrich, St. Louis, MO) and library preparations were performed according to the Pacific Biosciences 20 kb protocol with a final size selection of 9 kb using BluePippin (Sage Sciences, Beverly, MA, USA). Sequencing was performed using the Pacific Biosciences RSII sequencer, P6-C4 chemistry with 360 minutes movie time and one single-molecule real-time (SMRT) cell (Pacific Biosciences, Menlo Park, CA). The sequences were subsequently assembled using HGAP v.3 (Pacific Biosciences, SMRT Analysis Software v2.3.0) with default settings at The Norwegian Sequencing Centre (https://www.sequencing.uio.no/). The Minimus2 software of the Amos package was used to circularize contigs and the RS_Resequencing.1 software (Pacific Biosciences, SMRT Analysis Software v2.3.0) for correction of bases after circularization. Annotation of the sequences was performed using the NCBI Prokaryotic Genome Annotation Pipeline^37^.

*E. coli* K71-77 was used as donor strain in conjugation assays for the transfer of the *bla*_NDM-1_-encoding plasmid pK71-77-1-NDM. A sample of 23 previously characterized plasmid-naïve antibiotic susceptible clinical uropathogenic *E. coli* from the ECO-SENS collection were employed as recipient strains^25^. To facilitate selection during conjugation experiments rifampicin resistant (rif^R^) mutants of the recipient strains were generated by plating 100 µL of overnight cultures onto Lysogeny Broth (LB) agar supplemented with 100 mg/L rifampicin.

### Plasmid conjugative transfer

*E. coli* K71-77 served as donor while the rifampicin-resistant ECO-SENS strains served as recipients for the conjugative transfer of pK71-77-1-NDM. Single overnight LB cultures of donor and recipient were diluted 100-fold in 10 mL of LB and incubated at 37 °C with shaking until exponential phase was reached (OD_600_ = 0.6). Donor and recipient strains (1 mL each) were mixed in a 1:1 ratio and incubated at 37 °C for 3 h. Overnight cultures were diluted and plated on LB while mating cultures were plated on LB supplemented with rifampicin (150 μg/mL) and ampicillin (100 μg/mL). Conjugation frequency was calculated as $\frac{Transconjugants}{Donors}$. Experiments were performed with three biological replicates.

The presence of pK71-77-1-NDM in transconjugants was assessed by PCR for plasmid replicon and carbapenemase genes (IncA/C2 and *bla*_NDM-1_) and antibiotic resistance profile using the EUCAST disc diffusion method^38^. Transconjugants were further PCR-screened for the IncFIA replicon to confirm that pK71-77-2, the other plasmid present in the donor strain, was not transferred to the transconjugants. Transconjugant identity was verified by RAPD-PCR-profiling^39^ and Sanger sequencing of the *fumC* allele^40^. Primers are listed in Table S1. For each isolate, one transconjugant was frozen and used for further experiments.

### Plasmid stability

One transconjugant of each isolate was propagated by serial transfer for ≈300 generations, in three biological replicates. Serial transfers were performed in 1 mL of LB by diluting the previous culture 100-fold every 12 h. Plasmid presence was analysed at ≈300 generations by patching 100 colonies obtained from LB agar onto selective LB agar (ampicillin 100 μg/mL). After ≈300 generations, five plasmid-carrying clones per replicate were verified by PCR for plasmid replicon and carbapenemase genes and antibiotic resistance profile as described above. Additionally, RAPD-PCR-profiling was performed for one transconjugant per replicate.

### Fitness assays

Relative fitness of unevolved transconjugants was determined for each isolate by measuring relative growth rates. Growth rates for plasmid-free and plasmid-carrying rifampicin-resistant ECO-SENS strains were assessed by measuring the optical density of monocultures at 600 nm every 10 min for a period of 24 h, at 37 °C on a VERSAmax microplate reader (Molecular Devices, LLC). For this, 250 μL of a 1:100 diluted overnight culture in LB were added to 96-well microtiter plates. Experiments were performed in three biological and three technical replicates. Growth rates were determined using GrowthRates software version 3.0^41^.

Growth rates for each biological replicate resulted from the average of the three technical replicates. Fitness was calculated as: $\frac{growth\,rate\,plasmid-carrying\,strain}{growth\,rate\,plasmid-free\,strain}$. We measured three additional biological replicates for strains exhibiting fitness values with >0.05 standard deviation. Values outside the interval [Q_1_ − .5 · (Q_3_ − Q_1_), Q_3_ + 1.5 · (Q_3_ − Q_1_)], where Q_1_ and Q_3_ are the lower and upper quartiles, were considered outliers and removed from the analysis. Shapiro-Wilk test was performed to assess normality of data.

### Analyses of genomes

We downloaded the 14830 *E. coli* assembled genomes available at ftp://ftp.ncbi.nlm.nih.gov/genomes/genbank/bacteria/Escherichia_coli/latest_assembly_versions/ in December 2018. Multilocus sequence typing (MLST) was determined with MLST version 2.0 (default settings, species = “ecoli”)^42^ for all genomes. Genomes identified as belonging to ST10, ST12, ST69, ST73, ST80, ST95, ST100, ST135, ST141, ST372, ST537, ST607, ST998, ST1161 or ST1230 were selected for further analyses (Table S2). Genomes which contained alleles with less than 100% identity or 100% coverage were discarded.

We used PlasmidFinder version 2.0 (default settings, database = “Enterobacteriaceae”)^43^ to search for plasmids in the previously selected genomes. Statistical analyses and graphic illustrations were performed in R^44^.

## Supplementary information

Supplementary information.

## Data Availability

All data generated or analysed during this study are included in this published article (and its Supplementary Information File). PacBio sequences have been deposited in GenBank under BioProject PRJNA547487.
