# Supplementary material for: Host dependent maintenance of a blaNDM-1-encoding plasmid in clinical Escherichia coli isolates
Source: Sci Rep. 2020 Jun 9;10:9332. doi: 10.1038/s41598-020-66239-8 (PMC7283256; doi:10.1038/s41598-020-66239-8)
Supplement: Supplementary file 1 — Supplementary information. [file 41598_2020_66239_MOESM1_ESM.pdf]

Supplementary Information

Host dependent maintenance of a $bla_{\mathrm{NDM-1}}$-encoding plasmid in clinical *Escherichia coli* isolates

João Alves Gama[1†], Julia Kloos[1†], Pål J Johnsen[1], and Ørjan Samuelsen[1,2*]

[1] Department of Pharmacy, Faculty of Health Sciences, UiT The Arctic University of Norway, Tromsø, Norway

[2] Norwegian National Advisory Unit on Detection of Antimicrobial Resistance, Department of Microbiology and Infection Control, University Hospital of North Norway, Tromsø, Norway

[†] These authors contributed equally to this work.

Table S1. Primer sequences used in the study

Target gene	Direction	Sequence (5' to 3')	Reference
IncA/C2	F	GAGAACCAAAGACAAAGACCTGGA	[1]
	R	ACGACAAACCTGGATTGCTTCCTT	[1]
IncFIA	F	CCATGCTGGTTCTAGAGAAGGTG	[1]
	R	GTATATCCTTACTGGCTTCCGCAG	[1]
$bla_{\mathrm{NDM-1}}$	F	CAGCAAATGGAAACTGGCGACCAA	In-house
	R	ACGGTGATATTGTCACTGGTGTGG	In-house
RAPD-PCR	#1283	GCGATCCCCA	[2]
	#1247	AAGAGCCCGT	[3]
fumC	F	TCACAGGTCGCCAGCGCTTC	[4]
	R	GTACGCAGCGAAAAAGATTC	[4]

Table S2. Total number of genomes retrieved for each sequence type (ST).

ST	No. of genomes
ST10	988
ST12	117
ST69	242
ST73	305
ST80	26
ST95	348
ST100	22
ST135	19
ST141	38
ST372	24
ST537	6
ST607	15
ST998	29
ST1161	3
ST1230	0

References

1. Carattoli, A., *et al.* Identification of plasmids by PCR-based replicon typing. *J. Microbiol. Methods* **63**, 219-228 (2005).
2. Madico, G., Akopyants, N.S. & Berg, D.E. Arbitrarily primed PCR DNA fingerprinting of *Escherichia coli* O157:H7 strains by using templates from boiled cultures. *J. Clin. Microbiol.* **33**, 1534-1536 (1995).
3. Venieri, D., Vantarakis, A., Komninou, G. & Papapetropoulou, M. Differentiation of faecal *Escherichia coli* from human and animal sources by random amplified polymorphic DNA-PCR (RAPD-PCR). *Water Sci. Technol.* **50**, 193-198 (2004).
4. Wirth, T., *et al.* Sex and virulence in *Escherichia coli:* an evolutionary perspective. *Mol. Microbiol.* **60**, 1136-1151 (2006).
